# Mercury contamination, a potential threat to the globally endangered aquatic warbler *Acrocephalus paludicola*

**DOI:** 10.1007/s11356-017-0201-1

**Published:** 2017-09-26

**Authors:** Aneta Dorota Pacyna, Carlos Zumalacárregui Martínez, David Miguélez, Frédéric Jiguet, Żaneta Polkowska, Katarzyna Wojczulanis-Jakubas

**Affiliations:** 1 Faculty of Chemistry, Department of Analytical Chemistry, Gdansk University of Technology, 11/12 Narutowicza St., 80-233 Gdańsk, Poland; 2 Fundación Global Nature, Corro Postigo, 1, 34337 Fuentes de Nava, Palencia Spain; 3 Iberian Ringing Group (GIA-León), C/ Daoiz y Velarde, 49 Bajo, 24006 León, Spain; 4 Department of Biodiversity and Environmental Management, University of León, Campus de Vegazana s/n, 24071 León, Spain; 5 Centre d’Ecologie et des Sciences de la Conservation, UMR 7204 MNHN-CNRS-UPMC-Sorbonne Universités, CP 135, 43 Rue Buffon, 75005 Paris, France; 6 Faculty of Biology, Department of Vertebrate Ecology and Zoology, University of Gdańsk, ul. Wita Stwosza 59, 80-308 Gdańsk, Poland

**Keywords:** Conservation, Feathers, Passerine, Mercury, Warbler

## Abstract

Mercury (Hg) contamination is considered a global concern for humans and wildlife, and although the number of studies dealing with that issue continues to increase, some taxonomic groups such as small passerine birds are largely understudied. In this paper, concentration of mercury in the aquatic warbler (*Acrocephalus paludicola*) feathers, a globally threatened passerine species, was examined. The concentration differences between two ages and sexes were investigated. The comparison of feathers taken on autumn migrants of two age categories act as a comparison of the species’ exposure within the two different areas (European breeding or African wintering grounds). The average Hg concentration for all sampled individuals [2.32 μg/g dw (range 0.38–12.76)] is relatively high, compared with values found in other passerine species. An age difference was found, with first-year individuals displaying higher mercury concentrations than adults. This indicates that birds are exposed to mercury pollution during the breeding season, i.e., in the continental floodplains of eastern Europe. The average Hg concentration in feathers grown on the breeding grounds was 3.88 ± 2.59 μg/g dw, closer to the critical value of 5 μg/g dw, which is considered to impair the health of individuals. The findings suggest that mercury pollution may constitute a threat so far neglected for the endangered aquatic warbler.

## Introduction

Mercury (Hg) is a widespread contaminant released into the environment by both natural processes like volcanic eruptions, rock weathering, atmospheric transport, re-deposition (Fitzgerald 1989), and anthropogenic activities (i.e., burning of coal, emissions from multiple industrial processes, incineration, and goods manufacturing), with significant predominance of the latter (Burger and Gochfeld 1997; Selin and Selin 2006). Since mercury potent toxicity may present a threat to humans and wildlife (Seewagen 2010), it constitutes a great global concern when released into the natural environments as a pollutant. Post 1970 international policy has assured the continuous interest in the amount of released mercury (Selin and Selin 2006), resulting in conventions such as The Minamata Convention on Mercury established in 2013 (Evers et al. 2016). Mercury is among the most extensively studied of all environmental pollutants in wildlife and laboratory animals (Burger and Gochfeld 1997; Seewagen 2010). However, while a good recognition of usual and anomalous patterns is necessary for a comprehensive understanding of potential threats faced by wild populations, our knowledge of mercury distribution varies greatly across habitats and animal taxonomic groups.

Birds are clearly sensitive to mercury contamination. Many direct effects of mercury burden have been recognized in behavior, neurology, physiology, and reproduction (Seewagen 2010). The majority of research, however, has focused on select groups (e.g., predators and species associated with aquatic environments) (Seewagen 2010). These groups are expected to be at a greater risk of exposure and accumulation—predators due to position in the food chain and aquatic species due to overall high mercury concentration in its prey (Stewart et al. 1999). Passerines are understudied subjects of eco-toxicological studies (Janssens et al. 2002; Seewagen 2010). However, the group is not beyond the risk of mercury contamination. A few reports on mercury concentrations in passerines indicate that the risk is not necessarily negligible (Table 1).

**Table 1:** Mercury concentration [μg/g dw] in wild passerines from Europe and Asia

Species	Number	Age class	Feather type	Hg concentr. mean ± SE and/or range	Study years	Study period	Study site	References
Great tit (*Parus major*)	185	First years, adults	Tail	1.83–3.13^a^ 0.24–0.84^b^	1997–1998	November–May	Belgium	Janssens et al. 2001
Great tits (*Parus major*)	386	Nestlings	Tail	0.12–1.86^a^ 0.21 ± 0.06^b^	1999	Breeding season	Belgium	Janssens et al. 2002
Great tit (*Parus major*)	80	Nestlings	Tail	0.07 ± 0.02^a^ 0.01 ± 0.009^b^	2000	Breeding season	Belgium	Dauwe et al. 2004
Great tit (*Parus major*)	28	Adults	Tail	<LOD-3.15	2002	November–December	Belgium	Jaspers et al. 2004
Great tit (*Parus major*)	70	Nestlings	Tail	0.43 ± 0.13	2009	Summer	Portugal	Costa et al. 2013
Great tit (*Parus major*)	13	Adults	Tail	0.51 ± 0.46	2009	Summer	Portugal	Costa et al. 2013
Yellow wagtail (*Motacilla flava*)	5	Adults	Wing	2.1*	2006	January–August	Italy	Leonzio et al. 2009
Cetti’s warbler (*Cettia cetti*)	10	Adults	Wing	1.5*	2006	January–August	Italy	Leonzio et al. 2009
Italian sparrow (*Passer italiae*)	9	Adults	Wing	0.2*	2006	January–August	Italy	Leonzio et al. 2009
Eurasian tree sparrow (*Passer montanus*)	10	Adults	Wing	0.1*	2006	January–August	Italy	Leonzio et al. 2009
Reed bunting (*Emberiza schoeniclus*)	10	Adults	Wing	0.9*	2006	January–August	Italy	Leonzio et al. 2009
White-eared bulbul (*Pycnonotus leucotis*)	3	Unknown	Tail	0.17 ± 0.005	2005	April–October	Iran	Zolfaghari et al. 2009
Common blackbird (*Turdus merula*)	3	Unknown	Tail	1.08 ± 0.005	2005	April–October	Iran	Zolfaghari et al. 2009
Eurasian magpie (*Pica pica*)	2	Unknown	Tail	0.15 ± 0.005	2005	April–October	Iran	Zolfaghari et al. 2009
Rook (*Corvus frugilegus*)	2	Unknown	Tail	0.14 ± 0.07	2005	April–October	Iran	Zolfaghari et al. 2009
Hooded crow (*Corvus corone cornix*)	19	Unknown	Random	0.09 ± 0.04^c^ 0.52 ± 0.16^d^	Unknown	Unknown (molted feathers)	Israel	Adout et al. 2007
Feral pigeon (*Columba livia*)	50	Unknown	Random	0.04 ± 0.01^c^ 0.09 ± 0.02^d^	Unknown	Unknown (molted feathers)	Israel	Adout et al. 2007
Aquatic warbler (*Acrocephalus paludicola*)	72	Immatures, adults	Tail	2.32 ± 2.31 (0.38–12.76)	2013	Autumn migration	Spain, France	Present study

*LOD* limit of detection

^a^Close to pollution source

^b^More distant from the pollution source (4 km or more)

^c^Rural

^d^Industrial area

*Value from a graph

Owing to the fact that feathers represent the main route for mercury excretion in birds (Ochoa-Acuña et al. 2002; Frias et al. 2012), it is possible to determine mercury concentration in a relatively non-invasive way. This is particularly important for studying endangered species, as even small body feathers may be sampled and analyzed (Burger and Gochfeld 1997; Jaspers et al. 2004; Adout et al. 2007). Another advantage of feather samples is linked to the process and timetable which they are produced and replaced by birds. Trace elements are excreted into feathers during its growth, when the feather is fully grown its connections with blood vessels disappear, so that the feather becomes isolated from any further element uptake (Jaspers et al. 2004). This means that the feather represents a source of information about mercury presence in blood during the time of the feather growth (Jaspers et al. 2004). Birds replace their feathers according to species-specific schedules, with most migratory species molting particular feathers at different life stages and/or in different geographical areas. Appropriate feathers may then serve as a source of information about a bird’s environment during identified life-time stages.

Male and female birds are usually exposed to contamination at a similar extent, although adult females may transfer contaminants to eggs and therefore can have lower mercury concentrations compared to males (Burger et al. 2008). For this reason, it is important to consider the sex of birds, to fully comprehend a variation in the pollutant concentration. Numerous studies performed on monomorphic species neglect the sex issue due to technical problems with establishing the birds’ sex. Nevertheless, owing to the modern molecular techniques (based on a small amount of DNA) and/or discriminant functions (based on morphological measurements), the sex of birds can be efficiently established and can be considered in further analyses.

In this study, we aim to determine levels of mercury contamination in the aquatic warbler (*Acrocephalus paludicola*), based on feathers sampled from individuals captured along their migration flyway in late summer (autumn migration). The aquatic warbler is a small bird of particular conservation status, being the only globally threatened passerine species to breed in continental Europe (Salewski et al. 2013; Miguélez et al. 2014). Its population size and range have declined by ~ 90% over the last century, mainly due to habitat loss (Julliard et al. 2006; Flade and Lachmann 2008). Although the current population size is considered relatively stable with 22, 000–32, 000 adults (Briedis and Keišs 2016), the species is listed as vulnerable to extinction on the IUCN Red List (Birdlife International 2016). Habitat loss is undoubtedly the prime threat to the species, not only on breeding grounds but also on stopover migration sites and on wintering grounds (Birdlife International 2016). However, soil contamination by heavy metals (including mercury) could also threaten the fate of this species inhabiting floodplains in central and eastern Europe (St.Louis et al. 1994; Evers et al. 2012). Wetlands are a known hotspot for methylmercury, as their biogeochemical conditions tend to promote iron and sulfate-reducing microbes to convert inorganic mercury to methylmercury, a more toxic form than inorganic mercury (Ullrich et al. 2001; Ackerman et al. 2016). Litterfall could also represent a significant source of mercury, accounting for a high Hg (dry) deposition (Wang et al. 2016). Methylmercury accumulates in leaves and litterfall in terrestrial forests and wetlands and can be easily incorporated into the food chain. Consumed by herbivorous and detritivorous invertebrates, they present food sources for multiple bird species (Risch et al. 2017).

Since the aquatic warbler is a long-distance migrant, breeding in continental Europe and wintering in sub-Saharan west Africa (Julliard et al. 2006; Newton 2008; Jiguet et al. 2011; Salewski et al. 2013; Miguélez et al. 2014), it may be exposed to pollution across a large geographical area. Molt occurs once annually on the wintering grounds in Africa. In autumn, during migration, first-year individuals (i.e., birds hatched the same year and in full juvenile plumage, hereafter called immatures) possess only feathers grown in the nest on the breeding grounds. Older individuals (>1 year old; hereafter called adults) possess feathers grown the previous winter on the wintering grounds. The comparison of mercury contamination in feathers between these two age categories can then be considered as a comparison of the species exposure to mercury on the European (breeding) vs. African (wintering). This assumes that first-generation and older feathers have the same capacity to accumulate mercury.

## Materials and methods

### Study site

The birds were captured during their autumn migration (August to early September) at three stopover sites in Genêts (Mont Saint-Michel Bay, western France; 48° 41′ N, 1° 28′ W, season 2013, *N* = 8), Valcavado Stream (Zotes del Páramo, northwest Spain; 42° 15′ N, 5° 45′ W, season 2005, *N* = 17), and La Nava (Palencia, northern Spain; 42° 3′ N, 4° 45′ W, seasons: 2007 and 2008, *N* = 81). Mont Saint-Michel Bay is a wide littoral zone, extending over 500 km^2^ including 180 km^2^ of mud flats and 40 km^2^ of salt marsh (Laffaille et al. 2000). Valcavado stream is surrounded by areas covered with herbaceous and helophytic vegetation, temporarily flooded during summer (Miguélez et al. 2009). La Nava is a restored wetland classified as an important special protection area (Natura 2000; Muñoz-Adalia et al. 2015).

All birds captured were marked individually with a metal ring, measured (wing length) and aged according to plumage. The adults migrating in autumn, undergoing a complete molt on the wintering grounds, have a worn and abraded plumage, whereas immatures possess a new fresh plumage of only first-generation feathers (Svensson 1992). A single tail feather (occasionally two feathers) was collected from each individual to measure mercury concentration and determine sex based on molecular methods. Each feather was stored in a paper bag at ambient temperature until laboratory analyses.

### Mercury concentration

Each sample was processed using a standard procedure: carefully cleaned with deionized water and acetone to remove adherent external contamination (e.g., dust) and air-dried for 24 h. Mercury binds strongly to keratin functional groups and extensive washing with organic solvents such as acetone then do not significantly influence its content (Goede and de Bruin 1986, Jaspers et al. 2004). The whole, dry feather was then weighed (to the nearest 0.1 mg), cut into small parts (~ 2 mm^2^) using sterilized stainless scissors, and analyzed by thermal vaporization atomic absorption method (NIC MA-3000 Nippon Instruments Corporation). Samples were heat decomposed in a ceramic boat firstly heated up to 180 °C for 120 s and secondly to 850 °C also for 120 s. The mercury collector collects the atomized mercury gas in a form of gold amalgam, condensing and purifying the mercury. After heat decomposition, the mercury collection tube is heated to 650 °C to liberate the mercury gas. The absorbance at a wavelength of 253.7 nm is then measured. Oxygen flow was 0.4 L/min. The mercury concentration was determined in sample duplicate or triplicate (the amount of sample permitting), with the concentration expressed in micrograms per 1 g of dry weight of feather. Quality control includes blank samples every 8–9 subsamples run. The precision presented as the coefficient of variation of the concentrations of duplicates or triplicates of a single sample was 5.66 (range 0.00–14.69). Certified reference materials MODAS-4 Cormorant Tissue (M-3 CornTis), MODAS-3 Herring Tissue (M-3 HerTis), and MODAS-5 Cod Tissue (M-5 CodTis) were used to determine analytical accuracy and to perform method and quality control. The percentage recovery of the certified reference materials, measured on three replicates of each sample, varied from 94 to 100%. The limit of quantification was equal to 1.62 ng/g.

### Sexing

DNA was extracted from the basis of the collected feathers using a kit for forensic samples (Sherlock AX; A&A Biotechnology, Gdynia, Poland). We performed molecular sexing on feather-based DNA using a standard PCR-based method, with Multiplex PCR Kit (Quiagen, Germany), using F2/R1 pair of primers following Bantock et al. (2008) protocol. These primers target introns in ATP5A1—the alpha subunit of mitochondrial ATP synthase gene which represents a highly conservative unit of the genome (Bantock et al. 2008). The sex difference in intron length in PCR products was analyzed, with both pairs of primers clearly visible when separated on agarose gel (3.5%) stained with Midori Green Advanced DNA (Nippon Genetics, Japan) and viewed in UV light.

For a few individuals, the amount of extracted DNA was not sufficient to successfully amplify the target DNA region (21%), so we applied a discriminant function to determine their sex (Jakubas et al. 2014). The discriminant function estimated separately for adults (D_adults_ = wing length × 0.757 − bill depth × 5.009–31.889) and immatures (D_immatures_ = wing length × 0.824–52.473) allows for birds to be sexed with 87 and 75% probability of correct sexing for adults and immatures, respectively. Since bill depth (one of the two measurements in the function for adults) was not measured, the average bill length for adults migrating through France (3.02 mm) from Jakubas et al. (2014) was used. Three individuals remained unsexed due to missing measurements and an insufficient amount of DNA, and were excluded from the analysis.

### Data analyses

Based on the duplicate and triplicate samples, the variance coefficient of mercury concentration was calculated. If the coefficient > 15%, samples were excluded from the analyses, assuming unreliable estimation of mercury concentration. Of 106 samples, only 72 were further analyzed (23 adult males, 16 adult females, 17 immature males, 16 immature females). Mercury concentration was modeled in regard to age, sex, and age × sex interaction (fixed effects) using general liner mixed model with REML method [package *lme4*, Bates et al. 2015]. Since mercury concentration was measured multiple times for each individual, bird identity was included as a random effect to address potential problem of pseudoreplication. The response variable was log-transformed prior the analysis to meet assumptions of normality among residuals. To test significance of fixed effects, Wald tests were used. All of the analyses were performed in R (v.3.3.1, R Core Team 2016).

## Results and discussion

**Fig. 1:**
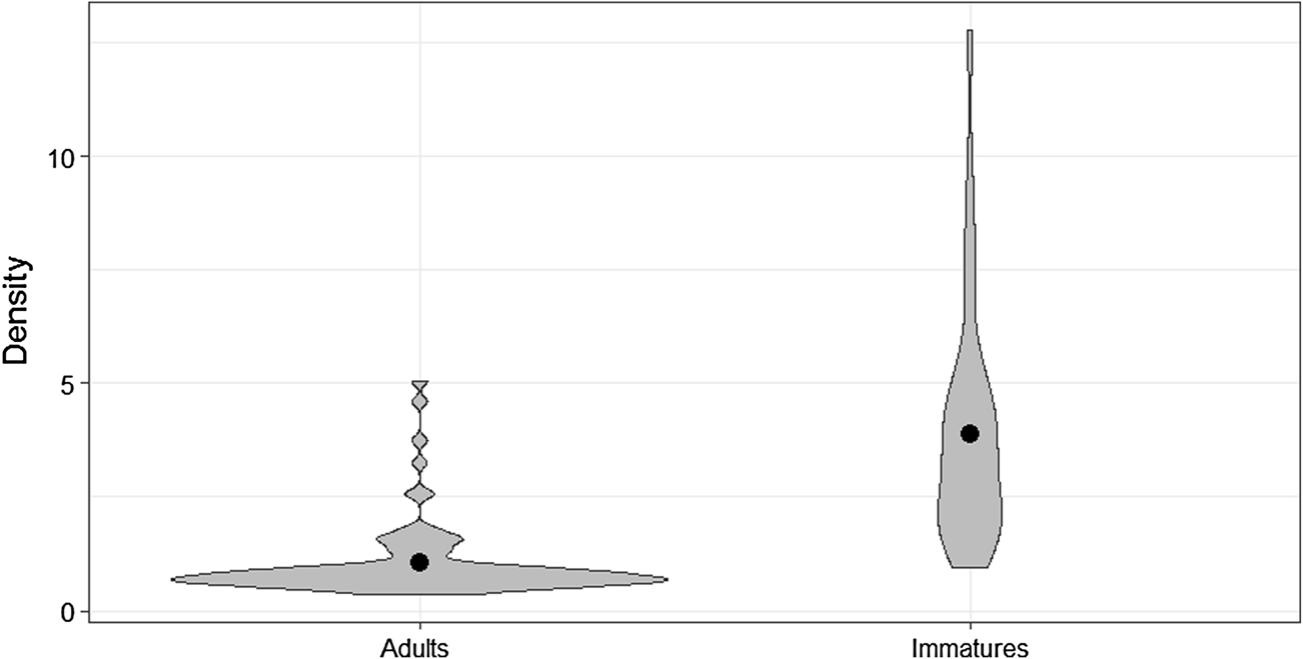
Distribution of mercury concentration [μg/g dw] in feathers of adults (feathers growing in wintering grounds in sub-Saharan West Africa) and immatures (feathers growing in continental floodplains of eastern Europe) of migrating Aquatic Warblers. Dots represent average values

A first report of mercury concentration in the aquatic warbler, feathers of 72 individuals, had an average concentration of 2.32 μg/g dw (95% CI 1.95–2.67 μg/g dw; range 0.38–12.76; Table 2). Of all explanatory variables, age was the most significant factor (LMM, *F*_1,68_ = 90.57, *p* < 0.001), with adult birds having significantly lower mercury concentration than immatures (Wald test, *t* = 5.45, *p* < 0.001; Fig. 1). The other two variables were of less important and insignificant (LMM, sex *F*_1,68_ = 1.57 *p* = 0.21; age × sex *F*_1,68_ = 2.05, *p* = 0.16).

**Table 2:** Mercury concentration (μg/g dw) of the aquatic warbler in regard to age and sex

Age	Sex	Number	Mean ± SD	Median	Minimum	Maximum
Immatures	Female	16	3.74 ± 1.11	3.49	1.11	9.88
Immatures	Male	17	4.02 ± 2.86	3.24	0.96	12.76
Adult	Female	16	1.31 ± 1.11	0.88	0.58	5.04
Adult	Male	23	0.84 ± 0.48	0.69	0.38	2.60

Average value for all studied individuals were lower than the critical value of 5 μg/g dw considered to impair the health of individuals (Burger and Gochfeld 1997). It is possible that Aquatic Warblers have a defense mechanism enabling them to detoxify and/or tolerate the toxic substance in the body. Rainio et al. (2012) found that migratory insectivorous bird species have high activity levels of the hepatic ethoxyresorufin-*O*-deethylase enzyme, while the enzyme’s activity is positively associated with the level of environmental contamination. Such a mechanism could evolve due to a wider range of natural toxins consumed by insectivore migratory species (Rainio et al. 2012). However, few individuals did exceed 5 μg/g dw (seven immatures and two adults, representing 12.5% of our sample), with the highest outliers at 12.7 μg/g dw. Also, comparing to other passerine species (Table 1), the values recorded for the aquatic warbler are notably high. Our findings indicate that aquatic warblers are exposed to the mercury contamination, with the contamination potentially representing a previously neglected threat for this globally endangered species.

Generally, older individuals tend to have higher residual contaminant concentrations in feathers due to bio-magnification processes, with numerous empirical studies showing age-specific differences in mercury concentration (e.g., Evers et al. 1998; Barbieri et al. 2010). Interestingly, here, immatures exhibited a higher mercury burden (Fig. 1, Table 2), with an average concentration of 3.88 ± 2.59 μg/g dw compared to 1.08 ± 0.79 μg/g dw in adults. However, the difference should be primarily interpreted in the context of spatially differentiated growing areas of the sampled feathers. Hence, the birds would experience higher exposure to mercury contamination on the breeding grounds (where feathers of individuals aged as first-calendar year in autumn have grown) across continental eastern and central Europe than on the wintering grounds (where all individuals in their second-calendar year or older replace all their feathers once a year at the end of the winter period) in sub-Saharian western Africa. Sampled individuals were captured during their autumn migration; consequently, we are not certain of their true breeding origin. However, recent work in France revealed that most of the world’s breeding population of aquatic warblers likely migrate by a western flyway over France and Spain (Jiguet et al. 2011). We can therefore consider that the sampled individuals likely originate from the largest breeding populations located in Poland, Belarus, and Ukraine. It is unclear whether the vast floodplains along main rivers, such as the Pripyat in Belarus or the Vistula in Poland, could have been contaminated by heavy metals more than the same kind of habitats in western Africa. However, this alone could explain the difference found here between European and African grown feathers and, as such, highlights the issue of the potential threat to the aquatic warbler on its breeding grounds.

We expected to find some sex differences in mercury concentration in the aquatic warbler, as, in general, adult females have a possibility to transfer contaminants to eggs and therefore may have lower mercury concentrations compared to males (Burger et al. 2008). However, we did not find a statistically significant effect of sex in both age groups explaining variation in the mercury concentration. This clearly shows that the processing of mercury contamination in wild species remains far from our understanding.

Where and how exactly aquatic warblers intake mercury remains unclear. Inhabiting and foraging in habitats such as marches and fen mires across continental Europe (BirdLife International 2016), that are far from both natural and anthropogenic sources of mercury, birds are not directly exposed to the contamination. However, birds may easily intake mercury through their diet, e.g., large insects developing in aquatic habitats (Kerbiriou et al. 2011), that in turn are believed to be usually good mercury source. Wetlands are known to be important natural sources of methyl mercury, with sites of net methyl mercury production (St.Louis et al. 1994). Also, due to global circulation system, biological mercury hot spots often do not occur close to mercury emitting sources (Evers et al. 2012).

## Conclusions

Our results indicate that aquatic warblers are exposed to mercury contamination, especially during the breeding season in continental Europe (average concentration for summer-grown feathers 3.88 μg/g dw, which is high compared with other passerines). The relatively high level of contamination suggests mercury pollution as a previously underestimated threat to this vulnerable species. We suggest that mercury concentrations should be measured in prey of the Aquatic Warbler at various breeding sites, to check if their food is the source of mercury contamination. We also suggest further studies on aquatic warbler populations to analyze temporal series and determine when mercury exposure may become a threat to the species. We hope our results will encourage researchers to examine the issue in other passerine species, as successful future bird conservation strategies depend on our understanding of various threats faced by endangered populations.
